# Vitamin D and its role in psoriasis: An overview of the dermatologist and nutritionist

**DOI:** 10.1007/s11154-017-9411-6

**Published:** 2017-02-07

**Authors:** Luigi Barrea, Maria Cristina Savanelli, Carolina Di Somma, Maddalena Napolitano, Matteo Megna, Annamaria Colao, Silvia Savastano

**Affiliations:** 1 I.O.S. & COLEMAN Srl, Acerra, 80011 Naples, Italy; 2 IRCCS SDN, Napoli Via Gianturco 113, 80143 Naples, Italy; 3 Dipartimento di Medicina e Scienze della Salute “Vincenzo Tiberio”, Rheumatology Unit, University of Molise, Via Francesco De Sanctis 1, 86100 Campobasso, Italy; 4 Dipartimento di Medicina Clinica e Chirurgia, Unit of Dermatology, Federico II University Medical School of Naples, Via Sergio Pansini 5, 80131 Naples, Italy; 5 Dipartimento di Medicina Clinica e Chirurgia, Unit of Endocrinology, Federico II University Medical School of Naples, Via Sergio Pansini 5, 80131 Naples, Italy

**Keywords:** Environmental factors, Vitamin D, Psoriasis, Dermatologist, Nutritionist, Nutrition

## Abstract

Psoriasis is a chronic immune-mediated inflammatory skin disease. Psoriasis lesions are characterized by hyper-proliferation of epidermal keratinocytes associated with inflammatory cellular infiltrate in both dermis and epidermis. The epidermis is the natural source of vitamin D synthesis by sunlight action. Recently, a role for vitamin D in the pathogenesis of different skin diseases, including psoriasis, has been reported. Indeed, significant associations between low vitamin D status and psoriasis have been systematically observed. Due to its role in proliferation and maturation of keratinocytes, vitamin D has become an important local therapeutic option in the treatment of psoriasis. To date, the successful treatment based on adequate dietary intake of vitamin D or oral vitamin D supplementation in psoriasis represent an unmet clinical need and the evidence of its beneficial effects remains still controversial. This information is important either for Dermatologists and Nutritionists to increases the knowledge on the possible bi-directional relationships between low vitamin D status and psoriasis and on the potential usefulness of vitamin D in psoriasis with the aim not only to reduce its clinical severity, but also for delineating the risk profile for co-morbidities cardiac risk factors that may result from psoriasis. In the current review, we analyzed the possible bi-directional links between psoriatic disease and vitamin D.

## Introduction

Psoriasis is a chronic immune-mediated inflammatory skin disease, with a prevalence of about 2%–3% in the general population [1]. The primary manifestation of psoriasis most commonly manifests on the skin, although inflammatory processes can occur also in other organs [1]. Indeed, nowadays psoriasis is considered a systemic pathology, including also other conditions, from psoriatic arthritis to obesity and metabolic disease (MetS), which increased cardiovascular risk in psoriatic patients [2–4]. Histologically, the dermatosis is characterized by hyperproliferation of keratinocytes, impaired epidermal barrier function at the sites of skin lesions, and skin infiltration by activated inflammatory cells [5]. The aetiology of psoriasis is not fully understood. Several factors contribute to its development, such as auto-immunological, genetic, hormonal and psychosomatic issues [6].

Vitamin D, also known as the sunshine vitamin, has long been known to be a hormone that regulates calcium-phosphorous homeostasis and safeguards the integrity of the skeletal system [7]. The epidermis is the natural source of vitamin D synthesis by the action of ultraviolet light (UV) B of the sun or other UVB source [8]. On the other hand, evidence is accumulating that vitamin D might represent a key modulator of immune and inflammation mechanisms [9]**.** Recently, a role for vitamin D in the pathogenesis of different skin diseases, including psoriasis, has been reported [9–11]. However, the effectiveness of vitamin D supplementation as adjunctive treatment option in psoriatic patients still remains controversial [9–11]. In the current review, we analyzed the possible bi-directional links between vitamin D and psoriatic disease.

## The dermatologist’s point of view

Patients suffering from psoriasis present a broad range of clinical phenotypes. Psoriatic lesions are classified into plaque, guttate, pustular, and erythrodermic types according to clinical features, especially regarding lesions size and distribution [1]. Disease onset may occur at any age, including childhood, with two peak age ranges, 16 to 22 and 57 to 60 years [2, 3]. Psoriasis lesions are characterized by hyper-proliferation with incomplete differentiation of epidermal keratinocytes and decreased keratinocyte apoptosis, associated with inflammatory cellular infiltrate in both dermis and epidermis [12]. Psoriasis Area and Severity Index (PASI) score is currently the preferred method for the assessment of the disease severity and extent [13].

### Effects of vitamin D on skin biology

The role of vitamin D as main regulator of skin physiology is very complex (Table 1). The epidermis is composed of four layers: basal layer (stratum basale), spinous layer (stratum spinosum), stratum granulosum and stratum corneum. The stem cells within the basal layer, which contact the basement membrane, continually divide during the lifetime of the organism, providing a source of cells which progressively migrate upwards through the epidermis, differentiating and stratifying to form the barrier layer of the skin [11, 14]. The process of epidermal differentiation is complex, sequential, and tightly controlled [11]. The precursor of vitamin D, 7-dehydrocholesterol, is located in the membranes of keratonocytes of the basal and spinous layer of epidermis [10]. By the action of UVB (wavelength between 290 and 315 nm), via a photochemical reaction, the B ring of 7-dehydrocholesterol is broken to form pre-vitamin D3 or cholecalciferol, which is subsequently converted first to 25-hydroxyvitamin D (25OHD) by the enzymes CYP27A1 and CYP2R1 and then to 1,25-hydroxyvitamin D (1,25(OH)D or calcitriol) the active form of vitamin D, by CYP27B1 [15]. Physiologically, the active form of vitamin D and its receptor regulate the differentiation and proliferation of keratinocytes, the balance of the cutaneous immune system and the process of apoptosis. The 1,25(OH)D has been shown to exert anti-proliferative effects on keratinocytes [16]. Numerous *in vitro* and *in vivo* studies have demonstrated dose-dependent effects of vitamin D on proliferation and differentiation of keratinocytes. Of interest, low concentration of vitamin D promotes keratinocyte proliferation *in vitro*, while at higher pharmacological doses a clear inhibitory effect became apparent [14, 17]. Moreover, 1,25(OH)D and analogs reduce S100A7 levels, generally up-regulated in psoriatic skin, in the reconstituted human epidermis stimulated by IL-22 [18], in interleukin (IL)-17-stimulated keratinocytes and in skin of patients with psoriasis [19]. Indeed, 1,25(OH)D regulates the cell proliferation in the stratum basale and increases the synthesis of keratins (K1 and K10), involucrin, transglutaminase, loricrin, and filaggrin, in the stratum spinosum [11, 14, 15]. Furthermore, vitamin D helps to regulate the synthesis of glycosylceramides needful for the barrier integrity and permeability in the stratum corneum [11, 14, 17]. These actions are due to the capacity of vitamin D to regulate intracellular calcium level, through induction of the calcium receptor, and the phospholipase C enzymes [19, 20]. A decrease or deficiency in 1,25(OH)D or a loss-of-function of its receptor has been shown to disrupt the differentiation of the epidermis, with reduced levels of involucrin and loricrin and loss of keratohyalin granules, resulting in hyperproliferation of the basal layer [11, 21–23].

**Table 1:** Vitamin D actions on skin

Vitamin D actions on skin biology and psoriasis pathogenesis
Regulation of keratinocytes proliferation, differentiation and apoptosis
Regulation of cutaneous immune system (inhibition of T cell proliferation, Tregs induction)
Down-regulation of pro-inflammatory cytokines
Stimulation of antimicrobial peptides expression
Regulation of barrier integrity and permeability

Vitamin D actions on skin biology and psoriasis pathogenesis

*Tregs,* Regulatory T cells

### Vitamin D regulation of apoptosis in keratinocytes

Calcitriol stimulates the synthesis of ceramide by inducing the neutral Mg^2+^-dependent sphingomyelinase (thereby increasing the conversion of sphingomyelin to ceramide) and in return, ceramide enhances the pro-differentiating effect of calcitriol on keratinocytes in a feedback loop [10, 24]. It has been demonstrated that physiological concentrations of calcitriol do not initiate apoptosis in cultured keratinocytes but, in contrast, pharmacological concentrations of calcitriol exert a pro-apoptotic effect on keratinocytes [25].

### Effects of vitamin D on the cutaneous immune system

Psoriasis pathogenesis implicates the innate and adaptive segments of the immune system. In particular, it is centrally controlled by T cells, in which an important role is played by T-helper (Th)1, Th17 and Th22, interplaying with numerous cell types via different cytokines, including tumour-necrosis factor-α (TNF-α), IL-6 and IL-17 [26]. The activity of these cells is modulated by specific T lymphocytes, named regulatory T cells (Treg) [9]. Regulatory T cells (Tregs) are able to inhibit the immunological response and to preserve the cutaneous immunological homeostasis, preventing autoimmune response against self-antigens [27].

There is an increasing interest on broad regulatory effects exerted by vitamin D on cells of the adaptive and innate immune system [28]. Indeed, vitamin D acts as a pluripotent immunomodulator that inhibits proliferation of T lymphocytes, induces generation of CD25+/CD4+ Tregs, a phenotype of T cells promoting tolerance and inhibiting immunity after stimulation with antigen. Moreover, vitamin D induces the expression of the C-C chemokine receptor type 10 on surface of T lymphocytes, a receptor involved in T cell-mediated skin inflammation, leading their migration from dermal blood vessels to epidermal kerotinocytes [29]. Finally, vitamin D helps to defend from opportunistic infections, by inducing autophagy in human macrophages, and to support the innate skin barrier, by stimulating endogenous antimicrobial peptides expression in resident epithelial cells of the skin [9]. Antimicrobial peptides, like cathelicidins and defensins, have not only properties against bacteria, fungi and viruses, but also other immune regulatory properties, including cytokine and chemokine release, antigen presentation, cell proliferation, increasing vascular permeability, angiogenesis and wound healing [30].

### Vitamin D and psoriasis linkage

In psoriasis, vitamin D is involved in the maintenance of cutaneous barrier homeostasis. Several studies identified an association between polymorphisms of vitamin D receptor (*VDR*) and psoriasis susceptibility [9]. Richetta et al., have found that the A-1012G promoter polymorphism of the VDR gene is associated with psoriasis risk through a lower expression of *VDR* mRNA, favoring conditions that may alter cutaneous barrier and the development of psoriatic lesions [31]. In addition, in psoriatic skin a decreased expression of *VDR* and reduced tight-junction proteins is associated [32]. Tight junctions are fundamental to regulate adhesion and permeability of keratinocytes, and to polarize cutaneous cell differentiation, to regulate extracellular calcium gradient, interacting with nuclear and cytoplasmic proteins and influencing the regulation of specific genes involved in keratinocytes differentiation and proliferation [32, 33]. Different studies have focused on the possible role of low vitamin D status in the pathogenesis of psoriasis [34–36].

Indeed, several studies reported that vitamin D is a key modulator of inflammation function [37, 38]. The active metabolite of vitamin D exert an anti-inflammatory effect on the inflammatory profile of human monocytes/macrophage [39–42], down-regulating the expression and production of several pro-inflammatory cytokines including TNF-α, IL-1β, IL-6, and IL-8 [43]. Moreover, dendritic cells differentiation, maturation, chemotaxis and antigen presentation seem to be dampened, and hydrogen peroxide secretion in human monocytes is also activated by 1,25(OH)D resulting in an increased oxidative burst potential [9, 44]. These anti-inflammatory effects support a role a low vitamin D status in the pathogenesis of psoriasis. Recent studies have shown that 1,25(OH)D values are significantly lower in psoriatic patients than in control subjects, even after adjusting for confounding factors in a multivariate analysis [11, 35]. In another study, vitamin D levels were lower in women with psoriasis in comparison with men, a difference not observed among controls [45]. Therefore, low levels of vitamin D are negatively associated with markers of inflammatory activation (C-reactive protein, CRP) and obesity [35]. Moreover, other studies showed that serum vitamin D levels were also reduced in patients with psoriatic arthritis and being inversely linked to disease activity [46, 47]**.**

### Topical vitamin D in psoriasis treatment

The beneficial effects of vitamin D induced by exposure to sunlight in the treatment of psoriasis have been known for decades. The effectiveness on psoriasis of vitamin D and its derivatives (calcitriol, calcipotriol, tacalcitol, hexafluoro-1,25(OH)D and maxacalcitol) have been known since 1985, being confirmed in numerous trials [45, 48]. The therapy with vitamin D, is one of the most popularly prescribed topical medications for this disease as the first-line, singly or in combination with topical corticosteroids, and numerous studies documented the efficacy and safety of using topical calcipotriol in the treatment of cases of localized plaque psoriasis [49–54]**.** Vitamin D analogs are particularly helpful for hard-to-treat areas such as the face or inguinal regions that are sensitive to steroid-induced atrophy [55]. Vitamin D analogs do not exhibit tachyphylaxis, as seen with corticosteroids, and topical treatment can be continued indefinitely without serious adverse side effects [9, 55]. Additionally, they are effective in the treatment of psoriatic skin lesions in children and elderly population [56–58]. A recent meta-analysis on the effectiveness of topical vitamin D therapies evidenced not only comparable efficacies to corticosteroids when used as monotherapy, but also superior effects when vitamin D used in combination with a potent topical steroid. According to these results, as topical vitamin D derivatives demonstrated a favorable safety profile, with “steroid-sparing” effects, and should be considered an indispensable component of the current physician’s arsenal in the treatment of psoriasis [11].

The therapeutic effects of topical vitamin D occur via a *VDR* mediated genomic mechanism resulting in inhibition of keratinocyte proliferation and mediated non-genomic mechanisms inducing keratinocyte differentiation by increasing intracellular calcium levels [59]. The anti-inflammatory effects may also result from inhibition of production of IL-2, IL-6, and interferon-gamma (IFN-γ). Further, topical calcipotriol inhibits human beta defensin and proinflammatory cytokines which are found in increased levels in psoriatic lesions [60]. Allelic variations in individual *VDR* genes may determine a different response to treatment: the isoform A of *VDR* is associated with a greater therapeutic response in psoriatic patients [61].

## The nutritionist’s point of view

Severe psoriasis has been associated with nutritional deficiencies because of an accelerated loss of nutrients, in particular of vitamin D, from the hyperproliferation and desquamation of the epidermal layer of skin [62–64]. Vitamin D supplementation is of particular interest to Nutritionists for two important reasons. First, besides its topical use, oral vitamin D supplementation represents an important adjunctive treatment option for psoriatic patients [9]; second, vitamin D supplementation might be very important for the prevention of psoriasis-related comorbidity [65], hypertension [66] and metabolic syndrome [67]**.**

### Food and vitamin D

There are two ways to meet vitamin D requirements in mammals: via nutrition and via synthesis in skin by from the sun or other UVB source [8]. Several recommendations were published regarding the dietary intake of vitamin D [68–70]. In particular, these recommendations they refer to dietary reference intakes for calcium and vitamin D updated by the Institute of Medicine (IOM) in 2010 [71]. To date, as the evidence for extra-skelatal effects of vitamin D are inconsistent and insufficient, the intake recommendations were based on beneficial effects of vitamin D only on skeletal health. The Recommended Dietary Allowances (RDAs) covering requirements of ≥97.5% of the population are shown in Table 2. Other organizations recommended different RDAs: the Endocrine Society suggested that adults aged 19–50 years require at least 600 international unit (IU) of vitamin D daily to maintain bone and muscular function [72]. However, the task force further annotated that 1500–2000 IU per day are necessary to consistently raise the serum level of 25(OH)D above 30 ng/mL. Finally, for older adults aged 60 and above, the International Osteoporosis Foundation (IOF) recommended a RDA of 800–1000 IU in order to reach a serum 25(OH)D level of 30 ng/mL [73].

**Table 2:** Vitamin D dietary reference intakes by life stage

Life-stage group	RDA (intake that covers needs of ≥ 97.5% of population)		Serum 25OHD level (corresponding to the RDA)*
	IU/d	mcg/d	ng/ml
Infants (0–12 months)	400**	10**	20
1–70 yr	600	15	20
+ 70 yr	800	20	20
Pregnant	600	15	20
Lactating	600	15	20

The Recommended Dietary Allowances (RDAs) covering requirements by life stage

*RDA* ***,*** Recommended Dietary Allowance; *IU* ***,*** International Unit

*Measures of serum 25(OH)D levels corresponding to the RDA and covering the requirements of at least 97.5% of the population

**Reflects adequate intake reference value rather than RDA. *RDAs have not been established for infants*

The diet is an important determinant of vitamin D status [71, 74]. Some studies have calculated that the amount of vitamin D intake that would ensure that the majority of the population (97.5%) maintains plasma 25(OH)D concentrations >25 nmol/l throughout the year is 8.7 mg/d [75]. Only few foods contain naturally vitamin D, and these foodstuffs are mainly of animal origin. The vitamin D status is the sum from the combination of synthesis in the skin after sun exposure and intake of the two main dietary forms of vitamin D: ergocalciferol (vitamin D2) and cholecalciferol (vitamin D3) [76]. The first is produced by plants, although fruits and vegetables in the human diet contain only minimal amounts of this nutrient, despite significant amounts of vitamin D2 are available through dietary supplements. The mushrooms provide variable amounts of vitamin D2, and if exposed to ultraviolet light under controlled conditions, the mushrooms can improve their content of vitamin D2 [77, 78]. The vitamin D3 instead, is naturally found in some animal foods, particularly in fatty fish, including salmon, herring, and mackerel, and in fish oils [79]. The fish (especially fatty fish and fish liver) have the highest natural content of vitamin D [80] Also egg yolk has a high vitamin D3 content [80], which strongly correlates with the content of vitamin D3 of the hen’s feed [77]. Depending on the hen’s diet [81] and UVB exposure [82], the vitamin D3 and 25(OH)D3 were transferred from the hen to the egg yolk. Regarding of meat products, the content of vitamin D depends on the contents of vitamin D in the fodder, the fat content of the meat product, and latitude where the animals have grazed [83]. Finally, the vitamin D3 is also available through dietary supplements, and is the form present in vitamin D-fortified foods such as milk, orange juice, and cereals. The fortification of food with vitamin D has been considered to be the most promising strategy with the broadest reach and impact [84–86]. In fact, in countries where this strategy was adopted proved to have a significant influence on the daily vitamin D intake in the average adult [87]. The food sources of vitamin D are listed in Table 3 [88].

**Table 3:** Food sources of vitamin D[88]

Food	Amount	IUs per serving**
Cod liver oil	1 tablespoon	1360
Swordfish, cooked	3 oz*	566
Salmon (sockeye), cooked	3 oz	477
Tuna fish, canned in water, drained	3 oz	154
Orange juice fortified with vitamin D (check product labels, as amount of added vitamin D varies)	1 cup	137
Milk, nonfat, reduced fat, and whole, vitamin D-fortified	1 cup	115–124
Sardines, canned in oil, drained	2 sardines	46
Liver, beef, cooked	3 oz	42
Egg (vitamin D is found in yolk)	1 large	41
Cheese, Swiss	1 oz	6

The food sources of vitamin D

*1 oz = 28,3495 g

**IU= International Units

### Bioavailability and influence of processing and cooking

The vitamin D, like other fat-soluble vitamins (A, E, K), is absorbed incorporated in mixed micelles from the intestine into the enterocytes by non-saturable passive diffusion. Subsequently, the vitamin D is transported in the chylomicrons via lymph to the circulation [89]. The more polar metabolite 25(OH)D is absorbed better and faster than vitamin D because it is also taken up directly from the proximal jejunum into the portal vein [79]. There are few data on its availability from natural sources. The absorption of vitamin D from supplements may differ depending on the used vehicle substance, such as oils, powders, ethanol [90]. For example, it has been reported that the bioavailability of vitamin D from fortified hard cheese is equivalent to supplements [91] and that vitamin D bioavailability is not influenced by the fat content of the fortified milk [92].

The cooking does not much influence the vitamin D content of animal foods. Mattila et al. [93] found that, in eggs boiled for 10 min, the vitamin D3 concentration was 1–6% lower and 25(OH)-D-3 content was 6–11% lower compared with raw eggs. In addition, also in fish, the cooking effect was moderate: baking various kinds of fish (e.g., perch, rainbow trout, Baltic herring) in the oven at 172 °C or 200 °C for 20 min induced a vitamin D3 loss of <10%, calculated on a dry matter basis [93]. The stability of vitamin D3 and 25(OH)D and vitamin D2 in foodstuffs during cooking has been shown to vary widely with heating process and foodstuffs, with reported retentions in eggs, margarine and bread after boiling, frying and baking of between 40% and 88% [94].

### The nutritionist and vitamin D supplementations

Supplements are the most important determinant of variation in vitamin D intake [85, 86, 95] A number of studies showed that the daily intake of vitamin D was higher in adults using vitamin D supplements than in those without vitamin D supplementations (348 IU vs 84 IU of vitamin D) [86]. Most nutritionists recommend the use of vitamin D3 to treat and prevent vitamin D deficiency, because several studies indicating a higher efficacy for vitamin D3 in raising serum 25(OH)D concentrations when compared to vitamin D2 [96]. Although a significant inter-individual variation exists, due to different variables including body weight, sunlight exposure and calcium intake, it has been calculated that supplementation of 1000 IU of vitamin D3 daily leads to an approximate increase in 25(OH)D levels by 10–20 ng/mL (25–50 mmol/L), [97–99]. Findings from randomised placebo-controlled trials conducted during the winter have shown that each 1 mg of supplemental vitamin D is associated with an increase in serum 25(OH)D of between 0.7 nmol/L [100] and 2 nmol/L [75].

Several studies have observed the safety concerns regarding the dosage of vitamin D supplementation. However, oral vitamin D intakes of up to 10,000 IU daily were not associated with any harmful effects [101], since this dose is comparable to the maximum cutaneous vitamin D production, and reports of vitamin D intoxication from cutaneous synthesis alone do not exist [101]. In this context, the IOM and the European Food and Safety Authority (EFSA) recommend a safe tolerable upper intake level of 4000 IU vitamin D *per* day for all adults, including pregnant and lactating women [102], although, to date, very few studies have investigated the long-term effects of a vitamin D intake above the suggested threshold of 4000 IU *per* day [103, 104]. To reach a steady state, after two to three consecutive months of treatment with vitamin D supplementation, it’s necessary a re-measurement of 25(OH)D serum levels [98].

Thus, the Nutritionists should consider a general vitamin D supplementation in populations at high risk for vitamin D deficiency, such as psoriatic patients [105]. The compounds of vitamin D commonly used in clinical trials varied from 1,25(OH)D, the physiologically active form of vitamin D, to 1αOHD, alfa-calcidol, requiring only liver metabolism to be converted to the active form to vitamin D or cholecalciferol, requiring both liver and kidney metabolism to become active. Perez et al. observed an overall 88% of clinical improvement of psoriasis with oral vitamin D with a decrease in mean PASI scores [61]. The results were confirmed in different studies on limited number of patients, reporting moderate or greater improvement in psoriasis in 25–50% of subjects [106–109]. It has also been proposed the therapeutic use of systemic alpha-calcidol in patients with psoriatic arthritis [61]. Another study demonstrated that a combination of acitretin and oral calcitriol resulted in a faster reduction of PASI in patients of chronic plaque psoriasis [110]. A study on a large population sample including 70,437 US females over a period of 14 years, examined the vitamin D intake levels and the incidence of psoriasis in the population. After adjusting for confounding variables, the study found that there was no significant association between vitamin D intake (dietary, supplementary, and total vitamin D) and the risk of incident psoriasis. Thus, the authors proposed that there was no role of dietary or supplemental vitamin D intake to prevent the development of psoriasis [111]. However, current recommendations for dietary intake of vitamin D are based only on its effect on skeletal health, while no information is present as regards psoriasis. In addition, a discrepancy between recommended RDAs and actual daily vitamin D intake exists. Therefore, solutions like food fortification and personalized diet or vitamin D supplementation in psoriatic patients need to be developed. In addition, taking into account the common association among psoriasis, obesity and Mets of which will be discussed in the next chapter, the advantage of the potential use of oral vitamin D supplementations to treat psoriasis and metabolic syndrome concurrently through its anti-inflammatory effects was strongly supported by a comprehensive meta-analysis including the results of clinical trials using vitamin D supplementation in psoriasis [106].

### Vitamin D, obesity and psoriasis

Many studies showed the link between psoriasis, obesity and MetS [112, 113]. Obesity is an important risk factor for psoriasis [114, 115]. The relationship between the two conditions is probably bidirectional, with obesity, mainly visceral obesity, predisposing to psoriasis and psoriasis favouring obesity [116]. In particular, there was a 2-fold increased risk for psoriasis development in the setting of obesity as compared with normal weight subjects [117]. In addition, for each unit increment increase in body mass index (BMI) was reported a 9% higher risk for psoriasis onset and a 7% higher risk for increased of PASI score [118]. A further evidence of the link between, obesity, inflammation and cardiovascular diseases in patients with psoriasis is provided by several studies reporting a correlation between PASI score and increased of CRP levels [119] and between psoriasis and waist circumference [120]. CRP is an acute phase protein significantly associated with obesity, representing the most sensitive markers of inflammation and an independent risk for cardiovascular disease. Waist circumference represents a surrogate measure of fat distribution highly correlated with visceral fat [121], the main source of inflammatory cytokines in obesity [122]. In particular, obesity and psoriasis are both pro-inflammatory conditions, in which the adipokines balance is shifted in favor to action pro-inflammatory adipokines [123, 124]. On the one hand, the dominance of pro-inflammatory adipokines favors either the development and the maintenance of obesity and its consequences, including cardiovascular diseases, type 2 diabetes, and MetS [125]; on the other hand, the production of inflammatory cytokines in visceral obesity represents the link involved in the complex mechanisms leading to the exacerbation of psoriasis and to psoriasis co-morbidities [126]. Indeed, psoriasis is frequently associated with cardio-metabolic co-morbidities and with an increased cardiovascular mortality [127, 128].

Similarly, there are significant associations between low vitamin D status and increased risk of obesity and obesity-related co-morbidities and cardiovascular mortality [127]. In particular, higher BMI leads to lower vitamin D status, with an inverse association between mortality risk and vitamin D levels, although this association could be indirectly mediated by the obesity per se [129]. Ju SY et al. performed a meta-analysis of the dose-response relationship between blood vitamin D levels and the risk of MetS. Using data on blood 25(OH)D levels, a monotonically decreasing relationship was observed for low levels of blood 25(OH)D in the pooled analyses of 16 cross-sectional studies (OR =0.89 for 30 nmol/L, OR =0.80 for 60 nmol/L, OR =0.71 for 90 nmol/L, and OR =0.63 for 120 nmol/L). A 25 nmol/L increase in 25(OH)D levels was associated with a 13% decrease in the risk of MetS in cross-sectional studies [130]. Several mechanisms might account for the low vitamin D status in obese subjects, [131]. Besides a low dietary intake of vitamin D linked to restrictive weight-loss diet regimen, the most likely explanations for low vitamin D status in obesity are sequestration of vitamin D in the adipose tissue [132], volumetric dilution related to the greater volume of distribution of 25(OH)D, less exposure of skin to sunlight due to less outdoor activity and air pollution [133]**.** The associations of low vitamin D status with obesity on the one hand, and with psoriasis on the other hand, lend support to the hypothesis for considering vitamin D as a further link between obesity and psoriasis. In this contest, a vicious cycle could operate among low vitamin D status, obesity, and psoriasis, with additive detrimental effects on cardio-metabolic risk in obese psoriatic patients. According to this hypothesis, vitamin D supplementation might be of particular usefulness for the prevention of psoriasis-related comorbidities.

## Conclusions

Due to its role in proliferation and maturation of keratinocytes, vitamin D has become an important local therapeutic option in the treatment of psoriasis. Indeed, significant associations between low vitamin D status and psoriasis have been systematically observed. Although the exact role of vitamin D in the pathogenesis of psoriasis is unclear, understanding the possible bi-directional relationships between low vitamin D status and psoriasis is also important for delineating the risk profile for co-morbidities that may result from psoriasis, such as obesity, type 2 diabetes, and MetS. To date, the successful treatment based on adequate dietary intake of vitamin D or oral vitamin D supplementation in psoriasis represent an unmet clinical need and the evidence of its beneficial effects remains still controversial. Nevertheless, the Nutritionists should consider a general vitamin D supplementation in populations at high risk for vitamin D deficiency, such as psoriatic patients. This information is important either for Dermatologists and Nutritionists to increase the knowledge on the potential usefulness of vitamin D in psoriasis with the aim to reduce not only its clinical severity, but also cardiac risk factors and psoriasis co-morbidities. Future well-designed dietary intervention trials with vitamin D supplementations on large population samples are needed to define the specific dose of vitamin D supplementations for psoriasis.

*1,25(OH)D*, 1,25-hydroxyvitamin D or calcitriol; *25OHD,* 25-hydroxyvitamin D; *BMI,* body mass index; *CRP,* C -reactive protein; *IL,* interleukin; *MetS,* metabolic disease; *PASI,* psoriasis area and severity index; *RDAs,* recommended dietary allowances; *TNF,* tumor necrosis factor; *VDR,* vitamin D receptor.
